# Role of Adipokines Signaling in the Modulation of T Cells Function

**DOI:** 10.3389/fimmu.2013.00332

**Published:** 2013-10-18

**Authors:** Claudio Procaccini, Veronica De Rosa, Mario Galgani, Fortunata Carbone, Claudia La Rocca, Luigi Formisano, Giuseppe Matarese

**Affiliations:** ^1^Dipartimento di Medicina e Chirurgia, Università degli Studi di Salerno, Salerno, Italy; ^2^Laboratorio di Immunologia, Istituto di Endocrinologia e Oncologia Sperimentale, Consiglio Nazionale delle Ricerche (IEOS-CNR), Napoli, Italy; ^3^Unità di Neuroimmunologia, IRCCS Fondazione Santa Lucia, Roma, Italy; ^4^Department of Biological, Geological and Environmental Sciences, Division of Pharmacology, University of Sannio, Benevento, Italy; ^5^IRCCS MultiMedica, Milano, Italy

**Keywords:** leptin, adiponectin, adipocytokines, T cells, obesity

## Abstract

The field that links immunity and metabolism is rapidly expanding. Apparently non-immunological disorders such as obesity and type 2 diabetes have been linked to immune dysregulation, suggesting that metabolic alterations can be induced by or be consequence of an altered self-immune tolerance. In this context, adipose tissue produces and releases a variety of pro-inflammatory and anti-inflammatory factors, termed “adipokines,” which can be considered as the bridge between obesity-related exogenous factors, such as nutrition and lifestyle, and the molecular events leading to metabolic syndrome, inflammatory, and/or autoimmune conditions. In obesity, increased production of most adipokines impacts on multiple functions such as appetite and energy balance, modulation of immune responses, insulin sensitivity, angiogenesis, blood pressure, lipid metabolism, and so on. This report aims to discuss some of the recent topics of adipocytokine research and their related signaling pathways, that may be of particular importance as could lead to effective therapeutic strategies for obesity-associated diseases.

## The Link between Adipose Tissue and Chronic Inflammation

It is well established from literature that in more affluent countries, where increased metabolic overload is more frequent, incidence of obesity is higher and it has been associated with a series of consequences, such as increased risk of cardiovascular disorders including atherosclerosis, diabetes, fatty liver disease, inflammation, and cancer (1–5). All these pathological conditions are closely associated with chronic inflammation, as they are characterized by abnormal cytokine production, increased acute-phase reactants such as C-reactive protein (CRP) and interleukin-6 (IL-6) and activation of a network of inflammatory signaling pathways. They seem to be consequent to the long-term “low-degree” chronic inflammation typical of obesity (6, 7).

A new field of study that investigates the interface and the link among immune response, nutrition, and metabolism has recently developed and many of the interactions between the metabolic and immune systems seem to be orchestrated by a complex network of soluble mediators derived from immune cells and adipocytes (fat cells) (8). It has been found that certain genetic alterations (i.e., mutation, loss of function, among others) of leptin (Lep), leptin receptor (LepR), pro-opiomelanocortin (POMC), pro-protein convertase 1 (PCSK1), and melanocortin-4 receptor (MC4-R), can cause obesity and can also significantly affect immune responses (9–16). Therefore, the immune function in obesity has become a factor of particular interest and relevance to better understand and possibly modulate the inflammatory condition associated with this disorder.

The current view of adipose tissue is that of an active secretory organ and not merely an inert tissue devoted to energy storage. Indeed it is able to send out and respond to signals that modulate appetite, energy expenditure, insulin sensitivity, endocrine and reproductive systems, bone metabolism, and inflammation and immunity (5). Recent studies have centrally placed adipose tissue as a crucial site in the generation of inflammatory responses. In this context, the finding that tumor necrosis factor-α (TNF-α) and IL-6 are overexpressed in the adipose tissue of obese mice and humans and when administered exogenously leads to insulin resistance, provided the first clear link between obesity, diabetes, and chronic inflammation (17–19). Moreover adipocytes share with a diverse set of immune cells (including T cells, macrophages, and dendritic cells) several features, such as complement activation, production of inflammatory mediators to pathogen sensing and phagocytic properties (20–22). In addition to adipocytes, adipose tissue also contains pre-adipocytes (which are adipocytes that have not yet been loaded with lipids), endothelial cells, fibroblasts, leukocytes, and most importantly, macrophages. Macrophage infiltration of adipose tissue has recently been associated with obese conditions and it has been suggested that expanding adipocytes or neighboring pre-adipocytes might be responsible for the production of chemotactic signals, leading to macrophage recruitment in the adipose tissue (23, 24). Once macrophages are present and active in the adipose tissue, they, together with adipocytes and other cell types present in the adipose tissue, might perpetuate a vicious cycle of macrophage recruitment and production of pro-inflammatory cytokines (25, 26).

Adipose tissue is a mix of adipocytes, stromal pre-adipocytes, immune cells, and endothelium, and it can respond rapidly and dynamically to alterations in nutrient excess through adipocyte hypertrophy and hyperplasia (27). With obesity and progressive adipocyte enlargement, the blood supply to adipocytes may be reduced with consequent hypoxia (28). Hypoxia has been proposed to be an inciting etiology of necrosis and macrophage infiltration into adipose tissue, leading to an overproduction of pro-inflammatory factors like inflammatory chemokines. This results in a localized inflammation in adipose tissue which propagates an overall systemic inflammation associated with the development of obesity-related co-morbidities (28).

There is increasing evidence that besides macrophages other immune cells, such as T cells, might infiltrate adipose tissue (29). Wu and co-workers recently presented evidence that, at least in mice, adipose tissue from diet-induced obese insulin-resistant mice is infiltrated by T cells and that this infiltration was accompanied by an increased expression of the T-cell chemoattractant RANTES (29).

The presence of an abundant immune cell infiltrate in adipose tissue of obese subjects is considered one of the classical pathologic lesions present in obesity. The real significance of these infiltrates is still unknown and has been until now, considered directly or indirectly the result of a massive attraction exerted by adipocytes toward immune cells, particularly of the natural immunity compartment (i.e., macrophages, neutrophils, natural killer cells, dendritic cells) through the secretion of adipocytokines and chemokines (30–32). Strikingly, a series of recent studies have shown in mice that T cells in the adipose tissue show specific T cell receptor (TCR) rearrangements suggesting that there are clonal T cell populations infiltrating adipose tissue. These data along with extensive macrophage infiltration and Th1 cytokine secretion account for the consequent insulin resistance in adipocytes and chronic inflammation typical of obesity (33). Taken together these data can lead to the hypothesis to consider obesity as an autoimmune disorder. Typically, criteria to consider a pathological condition as “autoimmune” include: (1) infiltration by immune cells of self-target organ and its consequent tissue damage; (2) the presence of circulating autoantibodies that react against self-antigens and subsequent complement system activation; (3) the clonality of TCRs from infiltrating T cells; (4) secretion of pro-inflammatory Th1 cytokines; (5) quantitative or qualitative alterations of regulatory T (Treg) cells; (6) association with other autoimmune disease. In the case of obesity, most of the above-mentioned points have been detected (34, 35). However the self-antigen present in the adipose tissue is still unknown. Identifying these antigens and the corresponding antigen-presenting cells in fat is clearly the next challenge for the field.

The discovery of leptin and other adipocytokines has provided a further link among adipose tissue and immune cells. These molecules, indeed, function as hormones to influence energy homeostasis and to regulate neuroendocrine function, but acting as cytokines, adipocytokines are able to module immune functions and inflammatory processes throughout the body. In this review, we provide an overview of recent advances on the role of adipocytokines and their signaling pathways in the modulation of immune cells function, with particular emphasis on T cells subsets.

### Leptin

Leptin, a cytokine-like hormone product of the obesity (*ob*) gene, belongs to the family of long-chain helical cytokines (characterized by a four a-helix bundle) and is mainly produced by adipose tissue, indeed its levels directly correlate with body fat mass and adipocyte size. However, it is produced, at lower levels, also by other tissues such as the stomach, skeletal muscle, placenta, and bone marrow (36–39). In the hypothalamus, leptin regulates appetite, autonomic nervous system outflow, bone mass, and the secretion of HPA hormones (36). Although an important role of leptin is to regulate body weight through the inhibition of food intake and stimulation of energy expenditure by increased thermogenesis, recent evidence has indicated that leptin is much more than a “fat sensor” (40). Indeed, leptin-deficient (*ob/ob*) mice and leptin-receptor-deficient (*db/db*) mice are not only severely obese, but also have a series of marked abnormalities that are secondary to the effects of leptin on reproduction (41), hematopoiesis (42), angiogenesis (43, 44), metabolism of bone (45), lipids and glucose (36), and last but not least, innate and adaptive immunity (46–48).

#### Leptin signaling

**Figure 1:**
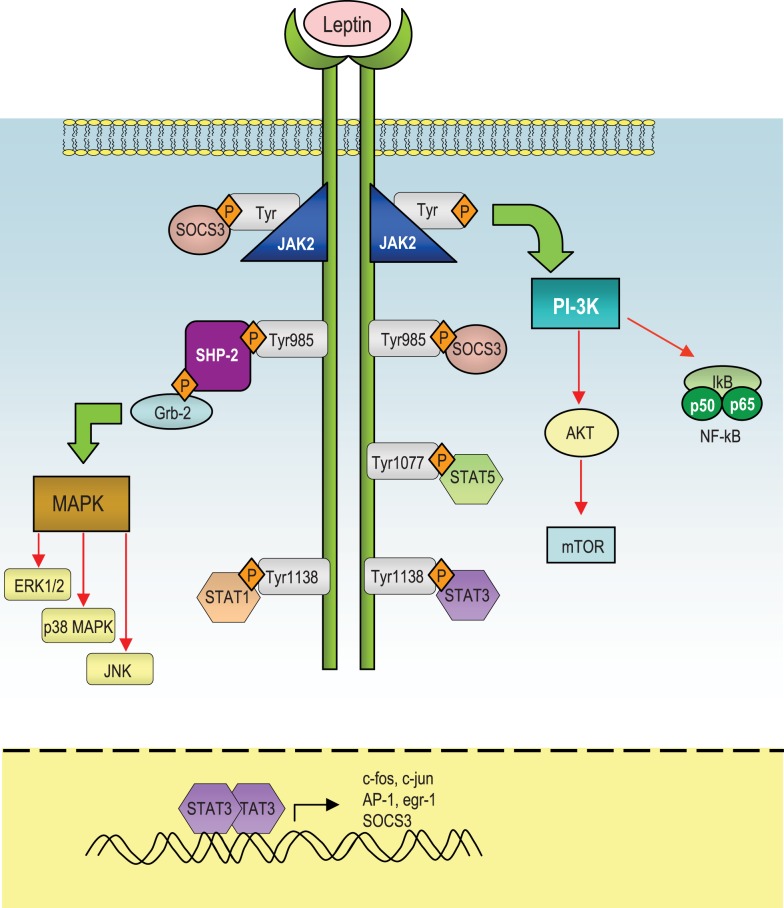
**Schematic representation of the leptin-induced pathways**. After leptin binds to the long isoform of the leptin receptor (LepRb), Jak2 is activated at the box 1 motif, resulting in the autophosphorylation of tyrosine residues and phosphorylation of tyrosines that provide docking sites for signaling proteins containing src homology 2 (SH-2) domains. The autophosphorylated Jak2 at the box 1 motif can lead to activation of phosphatidylinositol 3-kinase (PI3K)/Akt pathway. Akt can regulate a wide range of targets including FOXO1 and NF-κB. Both Tyr1077 and Tyr1138 bind to STAT5, whereas only Tyr1138 recruits STAT1 and STAT3. STAT3 proteins form dimers and translocate to the nucleus to induce expression of genes such as c-fos, c-jun, egr-1, activator protein-1 (AP-1), and suppressors of cytokine signaling 3 (SOCS3). SOCS3 negatively regulates signal transduction by leptin by binding to phosphorylated tyrosines on the receptor, to inhibit the binding of STAT proteins and the SH-2 domain-containing phosphatase-2 (SHP-2). SHP-2 activates the mitogen-activated protein kinase (MAPK) pathways including extracellular signal-regulated kinase (ERK1/2), p38 MAPK, and p42/44 MAPK through an interaction with the adaptor protein growth factor receptor-bound protein 2 (GRB2), to induce cytokine and chemokine expression in immune cells.

Leptin mediates its effects by the binding with the its specific LepR, a member of the class I cytokine receptor family (which includes receptors for IL-6, IL-12, OSM, and prolactin) and the pleiotropic biological effects of leptin can be partly explained by the wide distribution of LepRs on different types of cells, including those in extraneural tissues. Alternative splicing of LepR results in six receptor isoforms with different length of cytoplasmic domains, known as LepRa, LepRb, LepRc, LepRd, LepRe, and LepRf (49). Among all the LepR isoforms, only full-length isoform (LepRb) is able to fully transduce activation signals into the cell, as its cytoplasmic region contains several motifs required for signal transduction. The other LepR isoforms lack some or all of these motifs and their function is still unclear, even though several data suggest that they could be involved in the transport of leptin across the blood-brain barrier or in its degradation. Intracellularly, the LepR does not have an intrinsic tyrosine kinase domain, therefore it binds cytoplasmic kinases – mainly Janus tyrosine kinase 2 (Jak2) (50). LepR contains a highly conserved, proline-rich box 1 (51) and two putative, less conserved, box2 motifs (52, 53). Box 1 and box 2 motifs are considered important in recruiting and binding Jaks (54, 55) for full Jak activation (56). Recent studies indicate that, under physiological conditions, only Jak2 is activated during LepR signaling (53). Once activated, Jaks proteins trans-phosphorylate each other, as well as other tyrosine residues (Tyr985, Tyr1138, and Tyr 1077) of the LepR (57, 58), providing docking sites for downstream molecules such as signal transducer and activation of transcriptions (STATs). These proteins dissociate from the receptor and form homo- or hetero-dimers, which translocate into the nucleus and act as transcription factors by binding specific response elements in the promoter region of their target genes, such as sis-inducible-element (SIE), acute-phase-response-element (APRE), and GAS-like elements (59, 60) (Figure 1).

In response to leptin, STAT3 binds to phospho-Tyr1138, allowing Jak2 to phosphorylate and activate STAT3. Confirming the importance of this site of phosphorylation, mutation of Tyr1138 abolishes the ability of leptin to activate STAT3, resulting in severe hyperphagia and morbid obesity (61–63). Leptin stimulates also phosphorylation of LepRb on Tyr1077, which binds to STAT5 and subsequently mediates STAT5 phosphorylation (64, 65).

The Jak/STAT pathway is under the negative-feedback control of suppressors of cytokine signaling (SOCS) proteins, which are induced upon cytokine stimulation and act as negative regulators of signaling by binding to phosphorylated Jak proteins or by direct interaction with tyrosine-phosphorylated receptors (66, 67). Structurally, SOCS proteins are characterized by a central SH-2 domain, an N-terminal preSH-2 domain, in some cases a kinase inhibitory region (KIR) domain, which abolishes the kinase activity of the Jaks, and a more conserved C-terminal SOCS-box – which represents a key mediator of proteasomal degradation (by linking ubiquitin to the substrate) (66). Only SOCS1 and 3 carry a KIR domain in their N-terminal region and it is involved in the inhibition of the Jak activity and thus leptin signaling. Recent data showed that SOCS3 inhibits kinase activity through its KIR domain after the binding through its SH-2 domain with phosphotyrosine motifs in the receptor in the proximity of the Jaks. Interestingly, leptin can induce SOCS3 expression (68–71) and the Tyr985 of LepRb is a high-affinity binding site for SOCS3 (57, 70). In this context, the participation of SOCS3 in the negative-feedback mechanism of leptin signaling has been proposed to underlie the development of leptin resistance in relation to the hyperleptinemia observed during obesity (69).

Another negative regulator of leptin signaling is represented by the SH-2 domain-containing phosphatase-2 (SHP-2), which is a constitutively expressed tyrosine phosphatase involved in the dephosphorylation of Jak2 (72). SHP-2 carries two tandem SH-2 domains followed by a tyrosine phosphatase catalytic domain. When one SH-2 domain interacts with a tyrosine-phosphorylated ligand, a conformational change occurs and brings this phosphatase to activation of LepR at position Y985 (73). This specific site has an important role in leptin-induced extracellular signal-regulated kinases (ERK) activation (57). More specifically, as a result of leptin administration, Tyr985 becomes phosphorylated by recruited Jaks (mainly Jak2 and Jak1), and provides a docking site for SHP-2. After binding to that specific tyrosine residue, SHP-2 is phosphorylated at the C-terminus and together with its adapter molecule Grb2, it activates downstream signaling, leading to the activation of the p21Ras/ERK signaling cascade (57), with the final induction of specific target genes expression, such as c-fos or egr-1, a zinc-finger transcription factor that influences the initiation of growth and differentiation (74) (Figure 1).

Leptin can activate also another member of the MAP kinase family, p38 MAPK (75) and stress-activated protein kinase c-Jun N-terminal kinase (JNK). Among the possible downstream targets of leptin-induced activation of p38 and JNK MAPK pathways, the regulation of the transcription factor nuclear factor-κB (NF-κB) appears to be crucial for the transcriptional regulation of pro-inflammatory cytokines such as TNFα and IL-1β.

In addition, leptin is able to regulate phosphoinositide 3-kinase (PI3K) activity, indeed the binding of PI3K regulatory subunit to tyrosine-phosphorylated proteins induces a conformational change allowing the activation of its catalytic subunit and consequent full activation of PI3K, whose products typically stimulate protein kinases such as Akt, also called protein kinase B (PKB), protein kinase C (PKC) (76), and Forkhead box O1 (FOXO1), a transcriptional factor that is phosphorylated and inactivated by Akt (77–80). Leptin inhibits both the activity and expression of hypothalamic FOXO1 through the PI 3-kinase pathway (77). Indeed, overexpression of a constitutively active FOXO1 mutant decreases leptin sensitivity in mice with consequent increase in food intake and body weight, whereas small interfering RNA-mediated knockdown of FOXO1 increases leptin sensitivity and decreases food intake and body weight (77, 78).

Finally, leptin stimulates phosphorylation of ribosomal S6 kinase (S6K), a major physiological substrate of the mammalian target of rapamycin (mTOR) kinase in the hypothalamus. Indeed, rapamycin, a specific inhibitor of mTOR attenuates leptin’s anorexigenic effects (81), conversely, activation of S6K enhances leptin sensitivity (82) (Figure 1). mTOR binds to raptor and GβL to form the mTOR complex 1 (mTORC1), which directly phosphorylates and activates S6K (83). mTORC1 is inhibited by the TSC1/TSC2 complex (84–86). Akt phosphorylates TSC2 and inactivates the TSC1/TSC2 complex (85). Therefore, the mTOR/S6K pathway is likely to be a downstream target of the PI 3-kinase/Akt pathway in leptin-stimulated neurons.

#### Leptin and T cells

Leptin stimulates and promotes the proliferation of human peripheral blood mononuclear cells (PBMC) (40, 48), as the presence of LepR on monocytes and lymphocytes has been shown in mice (46, 87) and confirmed in human peripheral blood T-lymphocytes (both CD4 and CD8) (88).

In PBMCs, leptin stimulation induces tyrosine phosphorylation and translocation of STAT3 molecules to the nucleus (89–91) and the phosphorylation of the STAT3-associated RNA binding protein Sam68 (a tyrosine-phosphorylated adaptor protein in TCR activation, which is associated with the SH2 and SH3 domains of Src and other signaling molecules, such as Grb2, PLC-γ-1, and PI3K) (92–95).

Recent evidence has shown that leptin induces tyrosine phosphorylation of Sam68 and Insulin receptor substrate 1 (IRS-1), which associate with p85 (96, 97), the regulatory subunit of PI3K via the SH-2 domain, recruiting and leading to stimulation of PI3K activity (98). In this context, leptin has been shown to inhibit apoptosis of thymocytes through an IRS-1/PI3K-dependent pathway since this effect was inhibited by the PI3K inhibitor LY294002 (99). Moreover, Martín-Romero et al. have shown that both ERK-1 and ERK-2 were found phosphorylated in a dose-dependent fashion in PBMC after incubation with human leptin (98).

It was also found that leptin could induce sustained phosphorylation of p38 MAPK in human PBMCs and the phosphorylation of the ribosomal protein S6 – the only protein in the large 40S subunit that has been shown to be phosphorylated in response to growth factors and mitogens (100). One route of leptin-induced S6 phosphorylation in human PBMCs is via MEK and p42/p44 MAPK (101–103), which activate MAPK-dependent S6 Kinase p90 RSK and S6. The other way seems to be mediated via activation of p70 S6 kinase, since it has been shown that leptin phosphorylates p70 S6 kinase at Thr389 (104). Accordingly, pre-treatment of cells with rapamycin abolished this phosphorylation (104). Strikingly, the MEK inhibitor PD98059 has been shown to inhibit not only p90 RSK phosphorylation, as expected, but also p70 S6 Kinase and S6 phosphorylation, thus suggesting an essential role of MEK activation in a full induction of p70 S6 kinase activity in human PBMC (105, 106).

In CD4^+^CD25^−^ effector T cells (Teff), De Rosa et al. have shown that leptin-induced strong STAT3 phosphorylation, while stimulation of CD4^+^CD25^+^ Treg cells was not associated with a marked increase of phosphorylated STAT3 (107). SOCS3, a negative regulator of cytokine signaling, was activated by leptin blockade in Treg cells, in which the stimulation with anti-CD3/28 induced phosphorylation of ERK1/2 and subsequent cell proliferation (107). In the same subset of cells, the cyclin-dependent kinase inhibitor p27 (p27kip1, a molecule involved in the control of cell cycle and T cell anergy) was elevated before and after anti-CD3/28 stimulation, and leptin neutralization induced degradation of this molecule, partly explaining the reversal of the anergic state and proliferation of these cells.

Recently, the contribution of leptin to mTOR activation in human Teffs has been well defined. Indeed, it has been shown that leptin treatment had little effect on mTOR phosphorylation, but it induced a significant increase in p70S6K and S6 phosphorylation, concomitant with a consistent increase in AKT phosphorylation. The induction of mTOR, as well as AKT phosphorylation induced by TCR engagement, was significantly reduced by leptin blockade and this inhibition was partially reversed by the addition of recombinant leptin to cultures, thus suggesting suggest a link between autocrine secretion of leptin and mTOR activation in Teffs through an AKT-dependent mechanism (108). A recent study by Galgani et al. shows that nutritional status, through leptin, directly affects survival and proliferation of autoreactive T cells, modulating the activity of the survival protein Bcl-2, the Th1/Th17 cytokines, and the nutrient/energy-sensing AKT-mTOR pathway (109). Moreover, a paper by the same group has shown that leptin activates the mTOR pathway to control also Treg cells responsiveness (110, 111). More specifically leptin inhibited rapamycin-induced proliferation of Tregs, by increasing activation of the mTOR pathway. In addition, under normal conditions, Tregs secreted leptin, which activated mTOR in an autocrine manner to maintain their state of hyporesponsiveness. Finally, Tregs from db/db mice exhibited a decreased mTOR activity and increased proliferation compared with that of wild-type cells (110, 111). Together, these data suggest that the leptin-mTOR axis sets the threshold for the responsiveness of Tregs and that this pathway might integrate cellular energy status with metabolic-related signaling in Treg cells that use this information to control immune tolerance.

### Adiponectin

Human adiponectin is encoded by ADIPOQ gene localized on the chromosome locus 3q27. It has a sequence homology with a family of proteins characterized by an amino-terminal collagen-like sequence and a carboxy-terminal complement 1q-like globular region and shares homologies with collagens, complement factors, TNF-α, and brain specific factor cerebellin (112, 113). Two different forms of this molecule exist: a full-length protein, which is present in the plasma, and a globular adiponectin which consists of the globular C-terminal domain resulting from a photolytic cleavage mediated by a leukocyte elastase secreted by monocytes and/or neutrophils. After cleavage the globular form can trimerize, while the full length can exist as a trimer low molecular weight (LMW) adiponectin, as an hexamer, that consists of two trimers bound through a disulfide bond middle molecular weight (MMW) adiponectin and as a 12- to 18-mer high molecular weight (HMW) adiponectin. Adiponectin is mainly produced in white adipose tissue (WAT) by mature adipocytes, with increasing expression and secretion during adipocyte differentiation, but it can be also found in skeletal muscle cells, cardiac myocytes, and endothelial cells. Its levels inversely correlate with visceral obesity and insulin resistance and in this context weight loss is considered a potent inducer of adiponectin synthesis, thus suggesting a key role exerted by adiponectin in protection against obesity and obesity-related disorders. Indeed TNF as well as other pro-inflammatory cytokines such as IL-6 suppress adiponectin secretion in adipocyte (114, 115). Adiponectin acts thought the interaction with two different receptors: ADIPOR1 and ADIPOR2, which differ both in localization and binding affinity since ADIPOR1 is expressed mainly in skeletal muscle and binds globular adiponectin while ADIPOR2 is expressed mainly in the liver and engages the full-length adiponectin (116). Expression of ADIPORs has been reported on human monocytes, B-cells, and NK cells, but only a small percentage of T cells express these molecules (117). The binding of adiponectin to ADIPOR1 and/or ADIPOR2 results in the activation of peroxisome-proliferator-activated receptor-α (PPAR-α), AMP-activated protein kinase (AMPK), and p38 mitogen-activated protein kinase (MAPK). More specifically, AMPK acts as a major downstream component of adiponectin signaling, since it represents the cellular energy sensor in the body and it is normally activated when there is an increase in the intracellular AMP/ATP ratio (118, 119).

Over the past 5 years, several interacting and adapter proteins for ADIPORs have been discovered. The adaptor protein containing a pleckstrin homology domain, a phosphotyrosine domain and a leucine zipper motif (APPL1) has been shown to bind to ADIPORs (120, 121) and is required for adiponectin-induced activation of AMPK, p38 MAPK, and ERK1/2–MAPK pathways. In addition, the regulatory subunit of the protein kinase casein kinase (CK) 2 or the receptors for activated C-kinase-I (RACK-I) and the endoplasmic reticulum protein 46 (ERp46) have been reported as other potential binding partners for ADIPOR1.

Initial studies suggested that adiponectin could act as an anti-inflammatory adipocytokine, as it exerted its anti-inflammatory effects on endothelial cells through the inhibition of TNF-α-induced adhesion molecule expression (122). Adiponectin-deficient mice had higher levels of TNF-α expression in adipose tissue and higher plasma levels compared with wild-type mice (114). Adiponectin inhibited NF-κB activation in endothelial cells and interfered with the function of macrophages (122, 123), as testified by the finding showing that treatment of cultured macrophages with adiponectin markedly inhibited their phagocytic activity and their production of TNF-α in response to lipopolysaccharide (LPS) stimulation (123). Adiponectin increases the secretion of anti-inflammatory cytokines such as IL-10 and IL-1 receptor antagonist (IL-1Ra) by human monocytes, macrophages, and DCs and suppresses the production of IFN-γ by LPS-stimulated human macrophages (124) and Toll-like receptor (TLR)-induced NF-κB activation (125).

In addition adiponectin has been shown to be a negative regulator of NK cell function (77), since it suppressed IL-2-enhanced cytotoxic activity of NK cells through the AMPK-mediated inhibition of NF-κB activation and down-regulated IFN-γ-inducible TNF-related apoptosis-inducing ligand (TRAIL) and Fas ligand expression on these cells. Contrasting results have recently shown that adiponectin can also act as a pro-inflammatory cytokine. Indeed it has been shown that its levels are high in arthritis, preeclampsia, and end-stage renal diseases (126–130). Also, adiponectin was shown to induce production of the pro-inflammatory mediator IL-6 and activation of NF-κB in human synovial fibroblasts and adhesion molecule expression in endothelial cells (131–133). One possible explanation for the pleiotropic effects exerted by adiponectin could be the presence of various circulating oligomers of adiponectin. Although HMW multimers appear to be the most bioactive form of adiponectin in the circulation, other isomeric forms of adiponectin like hexamers could differently modulate intracellular signaling pathways in several anatomical districts, thus exerting quite different effects (134, 135). Thus, the question of whether adiponectin might be considered an anti- or pro-inflammatory adipocytokine still needs to be clarified.

#### Adiponectin and T cells

Little is know about the effect of adiponectin on T cell function. Several data suggest that adiponectin is a negative regulator of T cell activity. In particular, although a small percentage of T cells express ADIPOR on their surface, a great amount of T cells store ADIPORs within clathrin-coated vesicles and these receptors colocalized with Cytotoxic T-Lymphocyte Antigen 4 (CTLA-4) molecules. After stimulation of T cells, the expression of both ADIPORs and CTLA-4 has been shown to be upregulated. Interestingly, it has observed that the addition of adiponectin results in a significant decrease of antigen-specific T cell proliferation and cytokines production, through the enhancement of T cells apoptosis. Confirming these findings *in vivo*, adiponectin-deficient mice had higher frequencies of CD137^+^ T cells upon Coxsackie B virus infection, thus suggesting that adiponectin is a novel negative T-cell regulator (136).

Adiponectin has been shown to inhibit allograft rejection in murine cardiac transplantation, indeed Okamoto et al. have shown that allografts transplanted to APN^−/−^ mice showed severe acute rejection to transplants in APN^+/+^ hosts accompanied by increased accumulation of CD4^+^ and CD8^+^ T cells and macrophages (137). A recent paper by Tsang et al. suggests that the immunomodulatory effect of adiponectin on immune response could be at least in part mediated by its ability to alter dendritic cell functions (138). Indeed, adiponectin-treated dendritic cells show a lower production of IL-12p40 and a lower expression of CD80, CD86, and histocompatibility complex class II (MHCII). Moreover, in co-culture experiments of T cells and adiponectin-treated dendritic cells, a reduction in T cells proliferation and IL-2 production and an higher percentage of CD4^+^CD25^+^Foxp3^+^ Treg cells was observed (138) suggesting that adiponectin could also control regulatory T cell homeostasis. Moreover adiponectin inhibits the production of CXC receptor 3 chemokine ligands in macrophages and consequently reduces T-lymphocyte recruitment and accumulation during atherogenesis (139).

On the contrary, Cheng et al. have recently shown that addition of adiponectin to polyclonally activated CD4^+^ T cells induced secretion of IFN-γ and IL-6, increased phosphorylation of p38 MAPK and STAT4 and augmented T-bet expression, indicating that adiponectin enhances Th1 differentiation (140). In the same direction, the paper by Jung et al. has shown that adiponectin-induced maturation and activation of DCs, as demonstrated by the increased expression of MHC class II, co-stimulatory molecules in both mouse and human DCs, and it significantly enhanced production of pro-inflammatory cytokines. moreover, adiponectin-treated DCs significantly induced both Th1 and Th17 responses in allogeneic T cells, leading to enhanced pro-inflammatory responses (141).

### Resistin

Resistin is a 114-amino-acid polypeptide, originally shown to induce insulin resistance in mice (142). It belongs to the family of resistin-like molecules (RELMs), also known as “found in inflammatory zone (FIZZ),” a family of molecules that has been implicated in the regulation of inflammatory process (143). Resistin was shown to circulate in two distinct forms: a more prevalent HMW hexamer and a substantially more bioactive, but less prevalent, LMW complex (144). Initially, resistin has been shown to be predominantly expressed by adipocytes but recent evidence has suggested that macrophages, rather than adipocytes, appear to be the most important source of resistin in human subjects (145) and mRNA encoding resistin can be found in mice and humans in various tissues, including the hypothalamus, adrenal gland, spleen, skeletal muscle, pancreas, and gastrointestinal tract (146).

Contradictory findings have shown that resistin levels can be either increased, unchanged, or decreased in murine and human obesity and type II diabetes, however, recent data indicate that in human PBMCs, expression of resistin mRNA is markedly increased by the pro-inflammatory cytokines IL-1, IL-6, and TNF, and by LPS (147). Also, resistin levels are mutually correlated with those of cell-adhesion molecules such as intercellular adhesion molecule 1 (ICAM-1) in patients with obstructive sleep apnea, and in atherosclerotic patients are positively associated with other markers of inflammation, such as soluble TNF-R type II and lipoprotein-associated phospholipase A2 (148, 149). Similarly, stimulation of human macrophages with LPS led to increased resistin mRNA expression, via a cascade involving the secretion of pro-inflammatory cytokines and administration of LPS to human volunteers is associated with dramatically increased circulating resistin levels (150), thus suggesting that this molecule can act as a critical mediator of the insulin resistance associated with sepsis and possibly other inflammatory conditions. In further support of its pro-inflammatory profile, resistin also up-regulates the expression of vascular cell-adhesion molecule 1 (VCAM1), ICAM-1, and CCL2 by human endothelial cells and induces these cells to release endothelin-1 (151).

#### Resistin and T cells

A small number of studies have been performed to address the role of resistin in T cell functions, but recent evidence has showed that resistin strongly up-regulates the expression of TNF and IL-6 by human PBMCs and induces arthritis after injection into the joints of healthy mice (152). These pro-inflammatory properties of resistin were abrogated by an NF-κB inhibitor, thus showing the key role of NF-κB in resistin-induced modulation of inflammatory reactions. Moreover Son et al. have recently shown that resistin induces expansion of functional Tregs, as testified by increased protein and mRNA expression of FoxP3, only when CD4^+^ T cells are co-cultured with DCs (153).

### Visfatin

Another protein clearly representing an additional link between adipose tissue and inflammation is Visfatin [also known as pre-B-cell colony-enhancing factor (PBEF)] which has recently been identified as an adipocytokine secreted primarily by adipocytes in visceral fat and able to decrease insulin resistance (154). This molecule is an insulin-mimetic adipokine, being able to bind and activate the insulin receptor without competing with insulin. Visfatin mRNA levels increase in the course of adipocyte differentiation, and visfatin synthesis is regulated by several factors, including glucocorticoids, TNF, IL-6, and growth hormone. Originally it has been identified as a growth factor for B lymphocyte precursors PBEF (155) and since its discovery it has been associated with several inflammatory disease states such as acute lung injury (156, 157). Indeed the presence of specific single nucleotide polymorphisms in the visfatin/PBEF gene, which decrease gene transcription rate, highly increases the risk of development of acute lung injury in septic patients (157).

Furthermore, expression of visfatin has been shown to be upregulated in activated neutrophils from septic patients (155, 157) and to inhibit the apoptosis of neutrophils, through a caspase 3- and caspase 8-mediated mechanism (155). On monocytes, visfatin is able to induce their chemotaxis and their ability to induce allo-proliferative responses in lymphocytes, through a p38 and MEK-dependent mechanism. More specifically, it ha been shown that visfatin up-regulates the production of the pro-inflammatory cytokines IL-1b, IL-6, and TNF-α (158), the expression of the co-stimulatory molecules CD80 (B7-1), CD40, and also of ICAM-1 and other co-stimulatory ligand that binds to LFA-1 (lymphocyte function-associated antigen-1), thereby promoting the activation of T cells (159). In this context, Moschen et al. have also shown that PBEF/visfatin is a potent chemotactic factor particularly for CD14^+^ monocytes and CD19^+^ B-cells (158).

### Adipsin

Adipsin (which in human subjects corresponds to complement factor D46) is the rate-limiting enzyme in the alternative pathway of complement activation (160). Adipsin, together with several other components of both the classical and alternative complement cascade, is primarily expressed by adipocytes in mice and by both adipocytes and monocytes-macrophages in human subjects (161). Adipsin levels are reduced in murine models of obesity but either increased or unchanged in obese human subjects (162).

## Intracellular Metabolic Pathways in the Control of Immune Functions

Recent evidence shows that the intracellular metabolic pathways, that sense environmental signals, such as nutrient availability, are able to control T cell function and differentiation, including Treg cell activity and immune tolerance pathways. This might represent a mechanism that allows immune cells to finely tune their response according to their metabolic competence.

In particular, mTOR, a serine-threonine kinase that can integrate signals from environmental nutrients and growth factors to control T cell proliferation and differentiation (163, 164), together with AMPK, its activator LKB1, the NAD^+^-dependent deacetylase Sirtuin 1 (SIRT1), and the Forkhead-box-o-family (Foxo) proteins, have been described as the dominant intracellular elements linking metabolism and self-tolerance. mTOR kinase, which can operate in two distinct signaling complexes (mTORC1 and 2) (165, 166), regulates different aspects of helper T (Th) cell differentiation and fate. Differentiation of naive CD4 T cells into Th1 and Th17 subsets is controlled in part by mTORC1 signaling an event dependent on the small GTPase Ras homolog enriched-in-brain (Rheb) (167). In contrast, conditional deletion of mTORC2 adaptor rictor protein impairs Th1 and Th2 cell differentiation, without altering Th17 differentiation or frequency of Treg cells, by promoting phosphorylation of PKB or Akt, PKC, and NF-κB (168). In Treg cells, mTOR is a negative regulator of TCR-dependent FoxP3 expression (169), of *de novo* Treg cell differentiation (170), and of Treg cell lineage commitment (171).

In this context, several biological molecules have been associated to the control of intracellular metabolic pathways; among these the adipocyte-derived hormone leptin has been shown to bring the gap between metabolism and immune cell tolerance. We have previously demonstrated that leptin can be produced by, and inhibits, the proliferation of Treg cells (107). Indeed, genetic deficiency of leptin (*ob/ob* mice) is associated with an increased percentage of peripheral Treg cells as compared to WT mice. These data are in agreement with recent reports showing that adipose tissue in normal individuals is a preferential site of accumulation of Treg (34). Their precise role in this tissue is still object of extensive investigation but what is clear is that in mice, diet-induced obesity (DIO) is associated with a body mass-dependent, progressive decline in the proportions of Treg cells in the visceral adipose tissue (VAT). In contrast, therapy with CD3-specific antibody (which promotes T cell self-tolerance through global, transient T cell depletion) normalized insulin resistance and glucose homeostasis, and selectively restored CD4^+^Foxp3^+^ T cell pools in VAT (74), by increasing IL-10 and Th2/regulatory-type cytokines (34, 35). Moreover Cipolletta et al. identified peroxisome proliferator-activated receptor (PPAR)-γ, the “master regulator” of adipocyte differentiation, as a crucial molecular orchestrator of VAT Treg cell accumulation, phenotype, and function (172). All these data indicate that leptin could represent the molecular link between obesity and reduced number/function of Treg observed in this condition and on the basis of these data, one could predict that leptin might interact with the mTOR pathway. Supporting this hypothesis, leptin increases mTOR activation and blocks proliferation of cultured TCR-activated rapamycin-treated Treg cells and Teffs (108, 110), thus modulating immune tolerance.

## Concluding Remarks

During the last decade, there has been a growing understanding of how host nutritional status and metabolism can affect the immune response. In this context, several adipocytokines, are able to participate in a wide range of biological functions that include glucose metabolism and CD4^+^ T-lymphocyte proliferation, cytokine secretion, and apoptosis, underlining the link among immune function/homeostasis, metabolism, and nutritional state.

The notion that adipose tissue was considered as “passive” source of energy in time of famine and starvation has been completely revisited and its major role in the control of “dominant” functions, such as immunity and metabolism, is providing novel insights into the pathogenesis of metabolic and autoimmune disorders.

Although many effects of these adipocytokines have been elucidated in recent times, the details of their signaling pathways need further investigation to understand how they are ultimately integrated. It will be also worthwhile to focus, in the future, on how adipocytokines signaling integrates with the intracellular cascades activated by other factors in the immune cells, since understanding the mechanism of action of these adipocytokines will soon be pivotal to the development of novel therapeutic approaches to obesity-induced inflammatory diseases.

## Conflict of Interest Statement

The authors declare that the research was conducted in the absence of any commercial or financial relationships that could be construed as a potential conflict of interest.
